# Dilemmas in the Treatment of Premature Infants at the Borderline of Viability

**DOI:** 10.5041/RMMJ.10066

**Published:** 2011-10-31

**Authors:** Arthur I. Eidelman

**Affiliations:** Department of Pediatrics, Shaare Zedek Medical Center, Hebrew University School of Medicine, Faculty of Health Sciences, Jerusalem, Israel; Ben Gurion University of the Negev, Beer Sheva, Israel

**Keywords:** Extreme prematurity, medical ethics, mortality, morbidity, neonatal mortality, viability

## Abstract

As more reports emerge of improved mortality and morbidity rates in infants born at the edge of viability, there may be need to reassess protocols and recommendations that encourage only comfort care for infants who are born at less than 24 weeks’ gestation. Analysis of those studies that report extremely poor survival of these infants reveals that, all too often, the results are measures of a self-fulfilling prophesy that reflects a predetermined non-aggressive global policy of no resuscitation and minimal investment in intensive care. Furthermore, little distinction is made between high- and low-risk infants of the same gestational age despite repeated studies that indicate that one can identify subpopulations that have as much as a 20–50% increased chance of surviving with little if any long-term neurodevelopmental impairment. Thus, the need to reassess current policies is discussed.

## INTRODUCTION

It is nearly 40 years since Duff and Campbell^1^ in their classic paper first raised the issue of the moral and ethical dilemmas faced by physicians in the then called “special care nursery”. Thirty years ago Schechner^2^ coined the phrase “How small is too small?” as a provocative challenge to physicians charged with the care of the extremely premature infants in the delivery room and newborn intensive care units (NICUs). Decades later, neonatologists are now caring for infants weighing as little as 400 g and born as early as 22 weeks’ gestation, in contrast to the birth weights and gestational ages of the infants that were the subjects of the discussions of these authors (i.e. less than 1,000 g and 28 weeks respectively). Additionally, the concept of size has been replaced by the issue of the “limits of viability”. However, despite these developments, or possibly because of them, the ethical dilemmas have not only remained but intensified.^3^

To most, the adage that “good ethics starts with good facts” should be the guiding principle in resolving these dilemmas. Unfortunately, as will be detailed below, good facts, as in the words of Lantos,^4^ “do not necessarily lead to a moral consensus”. Even more problematic is the confusion as to what is the basis for determining which data constitute facts or biologic certainty and which are products of value judgments, which, in turn, create facts. Further complicating any analysis is the wide variation in both the immediate and long-term outcome results reported by different institutions that precludes generalization and extrapolation to the individual case in hand. As a result, 40 years after Duff and Campbell, we are still functioning in a moral gray area. To understand better this situation this manuscript will selectively review recently published results regarding treating or not treating infants at the limits of viability. The manuscript will not provide a comprehensive review or meta-analysis of all the published results, but rather will highlight those reports that have contributed to the seeming continuing moral confusion.

## THE LIMITS OF VIABILITY

Serial data from the US National Institute of Child Health and Human Development (NICHD) Neonatal Research Network have traditionally served many physicians worldwide as an idealized expectation of outcomes, a “clinical gold standard”. The validity of these data has been based on the assumption that the selected university-affiliated academic medical centers in the Network had access to relatively unlimited resources and have been guided by uniform clinical protocols of care provided by the US government. In addition, as the data generated by the Network units reflect the combined experience of 20 university-based NICUs, it theoretically avoids the statistical trap of analyzing too small and/or too selective a population. Its outcome results, thus, in theory could then serve as a valid bench-mark for measuring the success of a given facility (or geographic area) in caring for the extremely immature preterm infant and, in turn, provide a statistical basis for antenatal consultations with parents in a decision-making process as to what care to provide these infants.

The most recent report of the Network^5^ presents the data of 9,575 infants born at a gestational age ranging from 22 to 28 weeks and at birth weights ranging from 401 to 1,500 g who were born between January 1, 2003 and December 31, 2007. Most importantly, 4,160 infants were born between 22 and 25 weeks (< 26 weeks). The most striking outcome reported was the finding that despite all the newer technologies and treatment protocols there was no improvement in mortality in the cohort of infants born in the period of 2003–2007 as compared to 1999–2000 (Table 1). Analysis by birth weight stratification of the same data set confirmed this observation.^6^,^7^ This observation regarding the lack of improvement since the mid 1990 in the survival of such extremely low-birth-weight infants was also noted in the analyses of the larger (362 NICUs) but less uniform Vermont Oxford Network Database.^8^ These “surprising” results, in turn, raised the question: If this is the best we can do, have we reached a biologic reality that reflects the limits of our scientific and technical capacity in improving the chances of survival of these extremely premature infants, particularly those who are born at less than 24 weeks of gestation?

**Table 1:** Survival data: NICHD* Neonatal Research Network.

**Gestational Age**	**1987–1988**	**1999–2000**	**2003–2007**
22 weeks	–	–	6%
23 weeks	–	–	26%
≤ 23 weeks	23%	24%	–
24 weeks	34%	59%	55%
25 weeks	54%	72%	72%

* NICHD, National Institute of Child Health and Development

Countering this concern is an analysis of the same data set, wherein a wide range of infant survival from institution to institution has been documented (Table 2). Such data challenge clinicians to identify those demographic factors and/or practice parameters that can account for such variation in outcome within a supposedly highly selective and uniform care network and suggest that there is still a potential for improvement. Additionally, population-based outcomes from other data sets, such as the one from the Israel Neonatal Network,^9^ have noted improved mortality rates for the period 2004–2006 at 23 weeks as compared to the period 1995–2003. Similarly, data from Sweden^10^ for the period 2004–2007 have indicated that the survival rates for infants born at less than 26 weeks’ gestation continue to improve (10% at 22 weeks, 52% at 23 weeks, and 66% at 24 weeks) far exceeding those of the NICHD Network. Most striking was the report from a single institution tertiary regional unit^11^ that the survival rate of infants born live at 22 week was 20% in the period 1998–2003 and increased to 40% in the period 2003–2008. For those born during this period (2003–2008), at 23 weeks the survival rate was 63%, at 24 weeks it was 81%, and at 25 weeks it was 89% (Table 3).

**Table 2:** Range if survival: NICHD* Neonatal Research Network range of survival (n=20).

**Gestational Age**	**2003–2007 Total Population**	**Range**
22 weeks	6%	0%–50%
23 weeks	26%	2%–53%
24 weeks	55%	20%–100%
25 weeks	72%	50%–90%

* NICHD, National Institute of Child Health and Development; n, number of institutions.

**Table 3:** Survival data: Alabama Regional NICU.*

**Gestational Age**	**1998–2003**	**2003–2008**
22 weeks	20% (11/55)	40% (20/50)
23 weeks	62% (59/95)	63% (51/81)
24 weeks	77% (62/81)	81% (82/101)
25 weeks	77% (75/98)	89% (94/106)

* NICU, newborn intensive care unit.

Multivariate regression analysis of the NICHD total population data set by Tyson^6^ has noted that factors other than gestational age have significant impact on the survival of the infant born at less than 26 weeks of gestation. The four factors that improve survival are female sex, administration of antenatal steroids, singleton birth, and an increased birth weight. Bader^9^ analyzed the data set from the Israel Neonatal Network and confirmed that gender (female), prenatal steroids, and birth weight impact (lower) the mortality rates independently of gestational age and calculated that these individual factors can affect mortality at a given gestational age by differences of over 20% and cumulatively by as much as 50%. Lee^12^ calculated that infants born at 22–25 weeks and who are in the highest-risk category (male gender, no antenatal steroids, multiple birth, and lower weight percentile) have a mortality rate of over 80%, while for the lower-risk infants it is less than 20%.

Given all the above data, what is one to do when confronted with an impending delivery at the limits of viability, i.e. 22–24 weeks? Whose data should serve as the reference point for discussions with the parents? Whose data are so biased by a self-fulfilling prophesy of poor survival that they reflect an arbitrary decision not to initiate intensive care in infants born earlier than a given gestational age? Whose data have not factored in weight, gender, or administration of antenatal steroids in the decision-making process?^4^,^13^ In fact, careful perusal of the published reports does not allow one to conclude that we have reached the biologic end of the line and that there is no more room for further improvement in the survival rate of these extremely immature infants, as in essence we have become prisoners of our own expectations.

## LONG-TERM MORBIDITY OUTCOME

To many, the decision-making in this moral gray zone has been primarily influenced by the published data as to the long-term neurodevelopmental outcome of the surviving infants and not mortality rates. Reports on follow-up data from the NICHD Network^14^ from two treatment epochs (E1: 1999–2001 and E2: 2002–2004) have noted that there was *no* improvement in early childhood outcome between the two periods (mirroring the lack of improvement in survival rates). In both periods there was comparable use of prenatal steroids (approximately 80%), and there was no significant difference in the percentage of multiple births or female infants. The rate of significant neurodevelopmental impairment at 18–22 months in surviving infants born at 23 weeks or less was similar in both epochs, 23.6% in E1 and 26.5% in E2, and at 24 weeks it was 14.6% in E1 and 14.2% in E2. Most importantly, the percentage of the surviving infants born at 24 weeks or less who were unimpaired or only minimally impaired was no different in both epochs and was only 22%.

As such, these data highlighting such a poor outcome have served for many as the basis for the global recommendation of restrictive care for the infant born before 24 weeks of gestation, i.e. limiting care to non-treatment and comfort care only. Unfortunately, the fact that such recommendations are unrelated to the various factors that significantly modify survival rates speaks of poor ethical reasoning. In addition the recent reports such as those from large single regional institution^11^ that have documented improved neurodevelopmental outcome in infants born at less than 24 weeks (Table 4) strongly support the concept that we have not reached any final end-point. Furthermore, the assumption that comfort care is to the benefit of such infants is further belied by a report that the impairment rate in the surviving infants who did not receive aggressive care was higher as opposed to the rate in those who did receive maximum intensive care.^15^

**Table 4:** Neurodevelopmental impairment (NDI): Alabama Regional NICU.*

**Gestational Age**	**1998–2003**		**2003–2008**	
	**Total NDI**	Severe NDI	**Total NDI**	Severe NDI
22 weeks	67%	33%	37%	37%
23 weeks	62%	47%	48%	9%
24 weeks	77%	42%	35%	13%
25 weeks	32%	18%	23%	7%

* NICU, newborn intensive care unit.

Underlying the concern for the long-term neurodevelopmental outcome in this population is the fundamental question: Is the neurodevelopmental impairment seen in these preterm infants a product of impaired development of the brain as result of leaving the normal neurotrophic intrauterine environment or a result of damage to the vulnerable and immature brain? Of interest, the rate of cerebral palsy, which most likely reflects hypoxic-ischemic and/or hemorrhagic damage to the brain, is lower in recent years as perinatologists and neonatologist have become more adept in minimizing tissue-hypoxic events. On the other hand, the rates of cognitive and behavioral abnormalities have not fallen as much, if at all. As these functions are dependent on critical cortical development and neuronal synaptic connectivity, this may reflect the biologic realty that the brain of the preterm infant in an extra-uterine life does not develop in the same way as in the fetus *in utero*.^16^–^18^ Others have noted that when one compares term-equivalent preterm infants to term control infants with MRI studies, preterm infants have global and regional decreases in cortical gray and deep gray matter, less myelinated white matter, and smaller corpus callosal areas. More recent studies using the newly developed imaging biomarkers such as diffusion tensor imaging, voxel-based morphometry, and functional MRI have enabled investigators to begin to distinguish between the effect of premature birth per se as opposed to the effect of injury. Most interesting has been the findings that the preterm infant uses alternative neural networks to compensate for this delay in maturation.^19^,^20^ The recent review of Ment^21^ summarizing the state of the art of imaging biomarkers in the study of the developing preterm brain indicates that we will now have tools to test and compare the newer neurotrophic therapies that can potentially enhance a more normal postnatal development and/or facilitate compensatory functioning of the brain of such preterm infants.

## CONCLUSION

Both mortality and morbidity data strongly suggest that we have not reached the limits of what we can offer the extremely immature preterm infant, and even those born at a gestational age of less than 24 weeks are not foredoomed to a life burdened with significant neurodevelopmental impairment. From the perspective of over 40 years it is clear that there is still a dynamic process unfolding of improved outcome, and thus we should not treat treatment protocols as if they are chiseled in stone. As Lantos has repeatedly emphasized, “protocols reflect values as well as facts, but values create facts”. Given that the facts of treating infants at the limits of viability are at best varied and still changing and reflect both poorly understood and subtle variations in care practices, the approach to the individual infant who is to born in this gray zone of less than 24 weeks should be individualized and reflect the additional variables discussed above (gender, birth weight, antenatal steroids, etc.).^22^,^23^ The ultimate parental decision should reflect a shared decision-making process guided by physicians who are truly up to date with the sometimes inconclusive data that are available and are cognizant of the potential for the future. Acknowledging the reality that there is a moral gray zone in these situations will be the best guarantee that moral progress will evolve and be made.

## Abbreviations:

NDI: neurodevelopmental impairment;
NICU: newborn intensive care unit;
NICHD: National Institute of Child Health and Development (US).
